# Establishment of a modified percutaneous CT-guided paraspinal intramuscular VX-2 squamous cell carcinoma dual tumor model in rabbits

**DOI:** 10.7717/peerj.11536

**Published:** 2021-05-28

**Authors:** Liangliang Meng, Husheng Shan, Xiaofeng He, Jiantao Zhou, Jingxiang Huang, Xin Zhang, Li Ma, Xiaodong Xue, Zhongliang Zhang, Yueyong Xiao

**Affiliations:** 1 Department of Radiology, the First Medical Centre, Chinese PLA General Hospital, Beijing, China; 2 Medical School of Chinese PLA, Beijing, China; 3 Department of Radiology, Chinese PAP Beijing Corps Hospital, Beijing, China; 4 Institute of Orthopaedics, Chinese PLA General Hospital, Beijing Key Lab of Regenerative Medicine in Orthopaedics, Key Laboratory of Musculoskeletal Trauma & War Injuries, Beijing, China; 5 Anesthesia and Surgery Center, the First Medical Centre, Chinese PLA General Hospital, Beijing, China

**Keywords:** VX-2, Rabbit, CT, Tumor, Model

## Abstract

**Background:**

The rabbit VX-2 tumor model is a commonly used transplanted tumor model and is widely used in surgical, radiological, and interventional studies. Most of the known tumor models for each site are single solid tumors. This study aimed to establish an accurate and stable intramuscular dual tumor model guided by computed tomography (CT).

**Methods:**

In this study, we compared three different inoculation methods to select the most appropriate dual tumor model. Six New Zealand White rabbits were used as tumor-carrying rabbits for tumor harvesting. Thirty rabbits were divided into three groups as experimental rabbits. Group A applied the tumor cell suspension method, in which the suspension was injected into the designated location with a syringe under CT guidance. Groups B and C used tumor tissue strips obtained in vivo or under direct in vitro vision. The tumor tissue strips were implanted into the designated locations using a guide needle under CT guidance. The differences in tumorigenic rate, the size difference between bilateral tumors, and metastasis between the three methods were compared.

**Results:**

It was found that group A obtained a 100% tumor survival rate, but the size of the tumor was more variable, and needle tract implantation metastasis occurred in 5 cases. In group B, tumor tissue strips were taken in vivo for implantation, in which one case failed to survive. Tumor tissue strips in group C were obtained in vitro under direct vision. The tumor tissue strips obtained in vitro by puncture using a biopsy needle in group C had a 100% tumorigenicity rate and stable tumor size. No significant needle tract implantation metastases were found in either group B or C. The variance of tumor size obtained in group A was significantly higher than in groups B and C. The variance of tumor size in group C was the smallest. Group C had high tumorigenicity and a more stable size and morphology of the formed tumors.

**Conclusion:**

The results showed that the method of obtaining tumor tissue strips using in vitro direct vision puncture and implanting them into the muscle with CT guidance and guide needles can establish an accurate and stable dual tumor model. This dual tumor model can provide substantial support for relevant preclinical studies.

## Introduction

The VX-2 cell line is an epithelial-derived malignant squamous cell induced by the Shope virus (Kidd & Rous, 1940; Rous & Beard, 1935). The VX-2 tumor model in New Zealand white rabbits has been used extensively in preclinical applications in bone tumors, liver, kidney, and lung cancer (Li et al., 2018; Pezeshki et al., 2015; Wang et al., 2017; Zhang et al., 2009). The histopathological characteristics of rabbits are similar to those of humans. Studies in rabbits can be better extended to humans, which is an important reason why the VX-2 tumor model is widely used in preclinical studies (Shiomi, 2009). VX-2 tumors usually proliferate rapidly in the first three weeks, leading to cystic degeneration and necrosis in the later stages (Liu et al., 2012).

Researchers generally choose the appropriate tumor model for each study, such as liver cancer and kidney cancer tumor models (Bar-Zion et al., 2017; Lee et al., 2009; Styn et al., 2012; White et al., 2015; Zhang et al., 2009). The tumor model for early-stage liver cancer is a surgical incision followed by implantation of a tumor mass into the liver tissue, which is traumatic, slow to recover, and has a high incidence of infection and mortality (Chen et al., 2004). The most commonly used implantation method in animal experiments is to take a quantitative injection of tumor cells at the logarithmic growth stage after culture. Still, in practice, this method often fails to obtain a tumor model of stable size and location (Lee et al., 2009). Choi et al. (2017) developed a novel technique for creating a solitary VX-2 lung tumor in a rabbit lung cancer model. The technique involves inoculating a lipiodol- and Matrigel-containing tumor cell suspension into rabbits using a small needle under real-time CT fluoroscopy guidance. The novel method is an easy and safe method for successfully forming VX-2 solitary lung tumors in rabbits. This model will help surgical practice and interventional radiology studies of lung cancers (Choi et al., 2017).

However, most of these established tumor models are aimed at single solid tumors, whereas in clinical work, it is often necessary to consider the impact of local lesions or treatments on the whole body or other sites. In this case, simple and easy-to-apply dual-tumor models are missing. This dual-tumor model is critical in the field of minimally invasive local treatment of tumors. Minimally invasive treatments for tumors include radiofrequency ablation, cryoablation, microwave ablation, and irreversible electroporation (Facciorusso et al., 2020; Ko et al., 2020; Sorokin et al., 2020). According to the literature, these minimally invasive treatments not only kill tumors locally but also have a more or less anti-tumor effect on other lesions in the distal compartment, known as the abscopal effect (Sidana, 2014; Soule, Bandyk & Matteo, 2018). Besides, some experiments require a simultaneous comparison of the impact of different surgical approaches or drug treatments on the same individual. Therefore, a suitable dual tumor model is essential for preclinical studies. This study will try to establish a dual tumor model using three different inoculation methods and compare their differences in tumorigenic rate, model stability, and metastasis to identify the most stable and suitable dual tumor model for relevant preclinical studies.

## Materials & Methods

### Experimental animals

A total of 36 adults New Zealand white rabbits were recruited in this experiment. Six of these were tumor-bearing rabbits used for harvesting, and the remaining 30 were used as animals for constructing VX-2 tumor models. Each rabbit weighed approximately 3−3.5 kg. All rabbits were females. All experimental animals are used and raised in strict accordance with the corresponding laboratory guidelines. The housing environment is clean grade. Each rabbit was individually housed in a rearing cage at the Experimental Animal Center of the PLA General Hospital. To reduce the effect of confounding factors, we placed all metal rabbit cages in the same rearing room. Rabbits were arrived one day before experiments and housed under ambient conditions (23 °C, 46% relative humidity, and a 12-h light/dark cycle), with free access to water and chow. All investigators and breeders in charge of the animals had the required qualifications. All animals were purchased from Beijing Jinmuyang Experimental Animal Breeding Limited Liability Company (License number SCXK2015-0005). This study was approved by the Experimental Animal Ethics Committee of the Chinese PLA General Hospital (Approval number 2019-X15-26). The experimental protocol was reported to the ethics committee before the start of the study. This study was elaborated according to ARRIVE guidelines (Kilkenny et al., 2012). All operations are performed with minimal suffering to the rabbits. Since the rabbits would have tumors in their bodies after the experiments were completed, this might lead to lung metastasis, respiratory failure, cachexia leading to death. Therefore, to avoid suffering caused by the tumors, we considered it necessary to euthanize the rabbits after completing the experiment (14 days after implantation).

### Guidance device

Philips Brilliance large aperture 16-layer spiral computed tomography (CT) Scanner in the Interventional Center of the PLA general hospital was used for image guidance during tumor inoculation process and follow-up tumor growth. Scanning parameters: tube current 300 mAs, tube voltage 120 kV, layer thickness 5 mm.

### Anesthesia method

All rabbits should be fasted 6 h before the procedure to prevent choking-induced asphyxia during anesthesia. A modified mixture of midazolam injection (5 ml: 5 mg, 0.15−0.25 mg/kg) and xylazine hydrochloride injection (1.5 ml: 40 mg, 4–7 mg/kg) was used for rabbit anesthesia, which was administered in the posterior thigh muscle. Based on previous experience, a smaller dose (approximately 1 ml) is required for rabbits receiving anesthesia for the first time.

### Tumor sources

**Figure 1:**
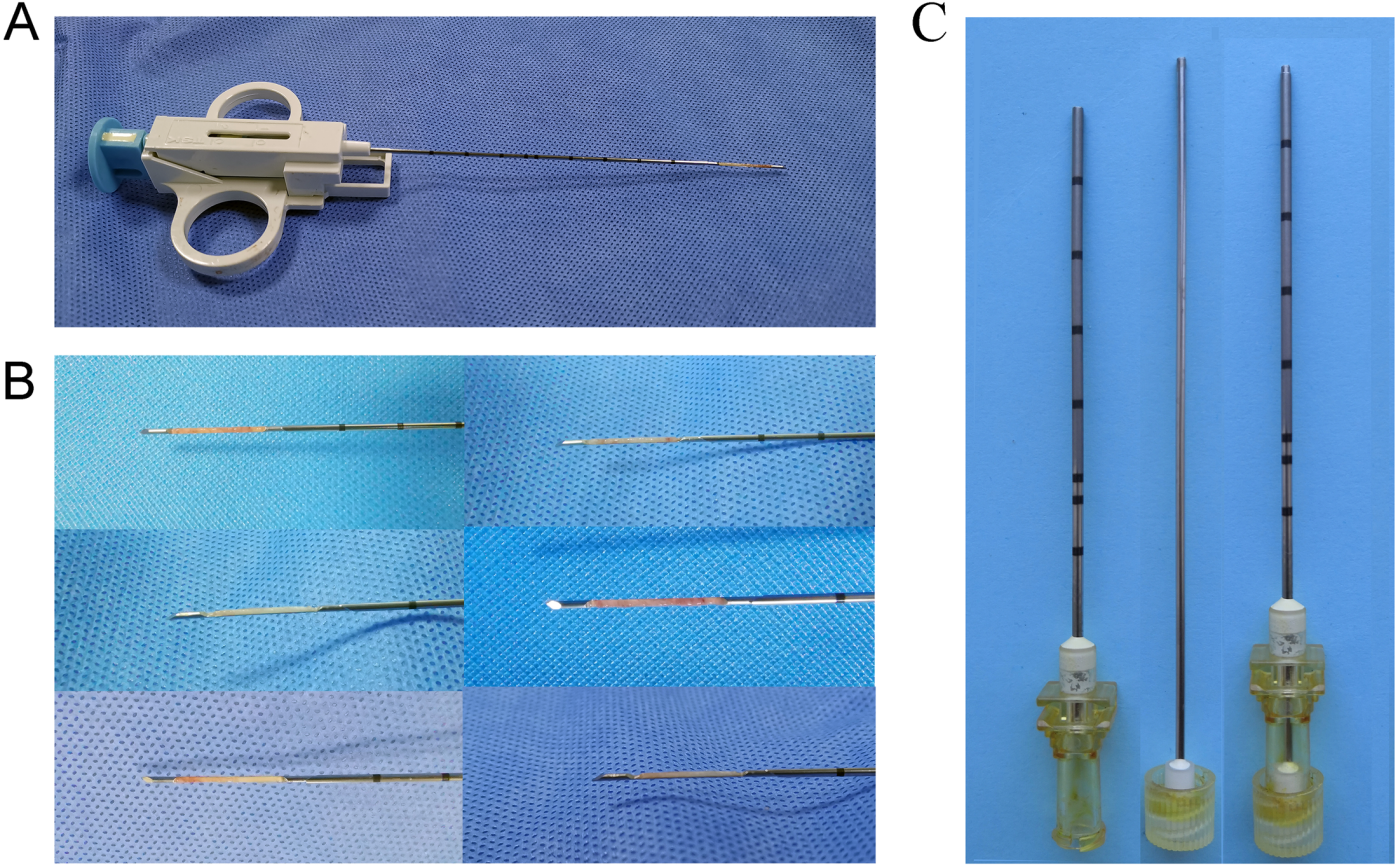
Preparation of tumor tissue strips. (A) An 18G semi-automatic biopsy gun was used to obtain uniformly sized strips of solid tumor tissue. (B) Tumor tissue strips obtained by in-vitro direct vision puncture biopsy. (C) 17G guide needle for implanting tumor tissue strips. The needle core tip was flattened to ensure the tumor tissue strip being pushed in as much integrity as possible.

The primary VX-2 cell suspension used in this study was obtained from the Institute of Orthopaedics in the Chinese PLA General Hospital. Remove the cryopreservation tube from liquid nitrogen, centrifuge the thawed tumor cell suspension, and prepare it for use. The concentration of cells was adjusted to 2.5*10^6^ cells/ml. After skin preparation and local sterilization with iodine and 75% alcohol, the thigh muscles of four rabbits’ hind legs were inoculated with 1 ml of tumor cell suspension for tumor harvesting. The same amount of cell suspension was injected into two other tumor carrier rabbits’ bilateral paraspinal muscles. The wounds were sterilized after the tumors were implanted. After awakening, the six rabbits were returned to the Experimental Animal Center for housing pending harvesting of the tumors. After about 20 days, the tumor tissue in the thigh and bilateral paraspinal muscles will grow to a diameter of 2.5–3 cm. Four of the rabbits with tumors planted in their thighs were euthanized, and the intact tumors were removed. After euthanasia, the rabbits’ fresh tumor was removed and rinsed by PBS buffer. We used the grinding method for tumor cell suspension preparation. The tumor cell suspension was prepared as follows: first cut the tissues into 1–2 mm size tissue pieces; put them into the tissue grinder, turn the rod and grind until homogenized; add 10ml of saline and rinse the grinder; harvest the cell suspension and filter it through 200 mesh nylon mesh, centrifuge at 1,500 rpm for 5 min; discard the supernatant, add red blood cell lysate to resuspend the sediment, act at room temperature for 5 min, add an equal volume of PBS to neutralize and then centrifuge at 1500 rpm for 5min, discard the supernatant, wash once with PBS, resuspend again with PBS and the cells are ready for use after counting under the microscope. The other two rabbits’ tumor masses will be used for biopsy under direct vision to harvest tumor tissue strips for implantation. An 18G semi-automatic biopsy gun (TSK Corporation, Japan) was used to obtain uniformly sized tumor tissue strips (Fig. 1A). Besides, two dorsally implanted carrier rabbits were anesthetized, and then CT-guided puncture biopsies were performed to obtain the required tumor tissue strips. Tumor implantation, as well as the process of harvesting, requires adherence to a strict aseptic regime.

### CT-Guided Implantation of VX-2 Tumors

**Figure 2:**
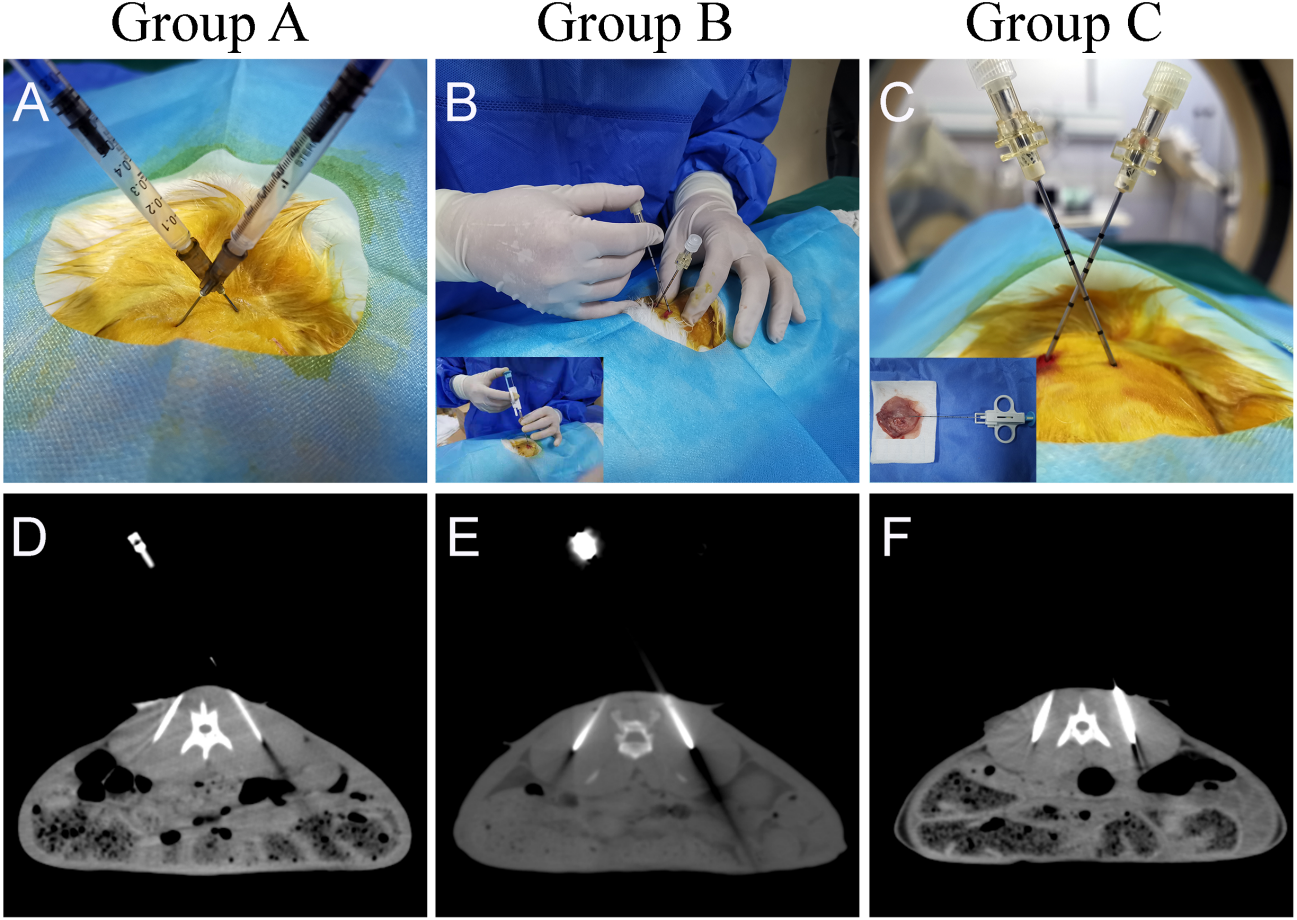
Different methods of tumor implantation. (A) Cell suspension injection was performed in group A. After the syringe needle was punctured into the designated location under CT guidance, 0.2 ml of tumor cell suspension was injected into the muscle. (B, C) The 17G cannula needle is used for the localization of the puncture. The 18G needle core was removed after reaching the designated position after CT scanning. The two tumor tissue strips were separately guided into the fixed implantation position with the specially flatted needle core. (D) CT images show that the syringe needle was inserted at the designated place in group A. (E, F) CT images show that the puncture needle sheath used to implant the tumor tissue strips has reached the implantation site. The arrows indicate the area where the tumor was implanted.

A total of 30 rabbits were randomly divided into three groups to establish a paraspinal intramuscular dual tumor model. Referring to previous studies, we included ten rabbits in each group (SM, S & LN, 2021; Xu et al., 2018). The tumor source for inoculation used in each group were different. Group A used the tumor cell suspension injection method, using a 1 ml syringe to inject 0.2 ml of the tumor cell suspension (5 ×10^5^ cells) into the designated locations as a control group (Fig. 2A). Ensure that the needle tip reaches the target location by local thin-layer CT scanning (Fig. 2D). In group B, an 18G semi-automatic biopsy needle and a matched 17G guide needle were used to harvest tumor tissues from in vivo rabbit tumors. In group C, the rabbit was first euthanized by air embolization, and the whole tumor tissue was removed with a scalpel after local sterilization. The diameter of the tumor was about 2.5–3 cm. The tissue strips were then obtained from the tumor tissue with a TSK semi-automatic coaxial biopsy needle under direct visual inspection. The strips’ integrity should be ensured as much as possible during the procedure (Fig. 1B). The central necrotic area should be avoided as much as possible during the puncture of the tumor tissue. Tissue strips with liquefaction and necrosis should be discarded. Tumor tissue strips removed with a puncture needle are relatively fixed in size, approximately 2 × 0.1 × 0.1 cm. The obtained tissue strips were then prepared in sterile 0.9% saline. Place the anesthetized rabbit in the prone position on the CT examination bed and place the localization grid after skin preparation at the puncture point. Select the center of the thickest part of the paraspinal muscle as the tumor site on the CT image. After routine sterilization and draping, a minor incision was made in the skin with a razor blade, and a 17G needle was passed through the incision to the designated location in the muscle. Because the 17G puncture guide needle is thicker and more difficult to puncture directly into the skin, so we would always make a minor incision in the skin with a blade to facilitate the entry of the guide needle. All groups do not require the muscle to be cut open. After the CT scan confirmed that the needle tips reached the bilateral target, the needle cores were removed (Figs 2E, 2F). The tumor tissue strips (Two strips for each side) in saline were placed into the punctured needle sheath, and the VX-2 tumor tissue strips were slowly pushed into the muscle with a matching homemade flatted needle core (Fig. 2). Group B and group C were identical in their inoculation methods. The difference lies mainly in the process of preparation of the tumor tissue strips. In group B, the tumor tissue was extracted by percutaneous puncture biopsy in vivo. At the same time, in group C, the animal was killed, and the whole tumor was removed, and a puncture biopsy was performed under visual inspection.

After surgery, all animals were returned to the housing room after regaining consciousness and resumed normal activities. The animal breeders should check the animals daily for abnormal signs of distress or complications. On days 7 and 14, the animals underwent an enhanced CT scan to observe the tumor’s growth. Enhanced CT images were obtained using 10 ml of iopromide injection, scanned immediately after a rapid push through the ear margin vein.

### CT imaging evaluation

After the tumor implantation was completed, a supine contrast-enhanced CT (CECT) scan was performed on postoperative days 7 and 14, respectively, to observe the tumor’s growth. After 10 ml of iopromide injection was rapidly pushed intravenously through the ear margin, a CT scan of the implantation site was immediately performed. Tumor size can be measured by CECT. To reduce the influence of parameters on measurements, we used the same parameters for all CT scans. Besides, indicators that should be monitored include syringe implants, pulmonary or abdominal metastases, etc.

### Pathology validation

On day 14, after implantation, three rabbits in each group underwent enhanced CT-guided puncture to obtain tumor tissue. The tumor tissues were fixed in formalin, dehydrated, embedded, and made into wax blocks, and then stained with hematoxylin-eosin (H&E) to observe the pathological manifestations of the tumor tissues.

After completing the experiment, to avoid suffering, rabbits were then euthanized by intravenous injection of an overdose of sodium pentobarbital.

### Statistical analysis

Statistical analysis was performed using SPSS software (IBM SPSS statistics version 25, Chicago, IL, USA). If the tumor size data in each group conformed to a normal distribution and chi-squared, an independent sample *t*-test was used to compare the two groups. Data with unequal variances were analyzed using nonparametric analysis. To compare whether there was a significant difference in tumor size between the two sides formed by different implantation methods, we used a one-sample *t*-test to compare the statistical difference between the difference in tumor size between the two sides of each group and different reference values, respectively. The reference values are set at one mm, two mm, and five mm. The statistical threshold was set at a *P*-value <0.05. The dual tumor model’s stability was mainly determined by comparing the variance of tumor sizes in each group, a one-sample *t*-test based on different reference values, and one-way ANOVA for the size difference between dual tumors in each rabbit.

## Results

### Tumor modeling

**Figure 3:**
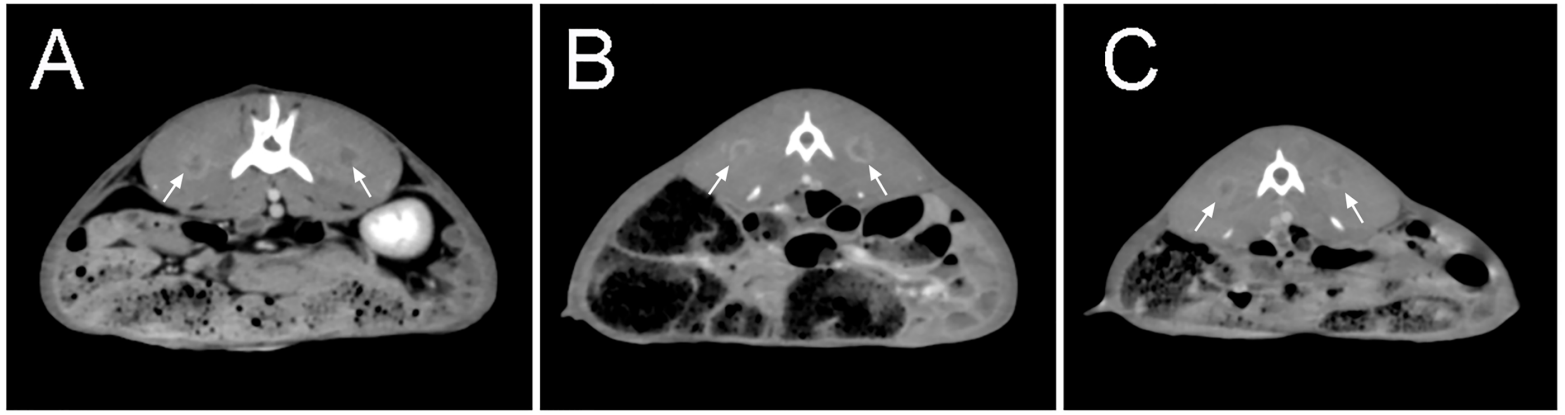
Enhanced CT scan at seven days after tumor implantation. (A–C) There was no significant tumor size difference among all groups. After enhancement, the tumors were small in size, with some showing small ring-shaped enhancement and some showing patchy and nodular shape. The arrows indicate the formed tumor lesions.

One week after tumor implantation, confirmed by enhanced CT scan, it was found that all tumors in group A were successfully implanted, with small flakes and smaller ring-like enhancements visible, with an average diameter of approximately 8.59 mm. No significant enhancement was found on one side of one rabbit in group B. The result on this side of the rabbit was not included in the statistical analysis. The results obtained in group C were similar to those in group A, with an average tumor diameter of approximately 6.91 mm (Fig. 3) (Table 1).

**Figure 4:**
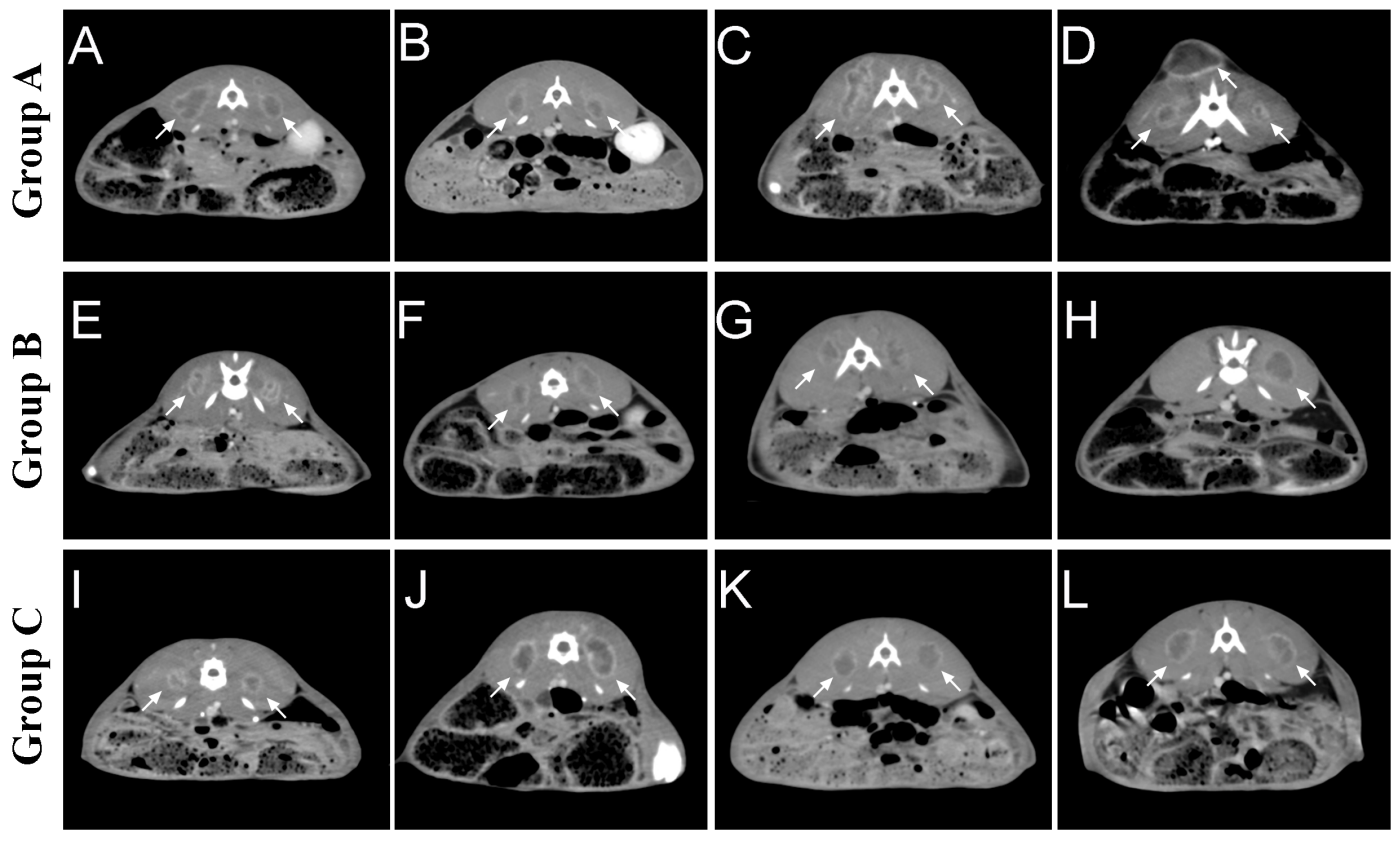
Tumor growth in each group two weeks after tumor implantation. (A–D) In group A, it was seen that all rabbits were successfully implanted with tumors, some with irregular morphology and individually visible tract seeding (C). (E–H) In group B, the morphology of the tumors formed using the tissue strip method was still regular, with individual implantation failures (H). (I–L) Group C used in vitro direct-view tissue strips, which were successful in tumor formation in all rabbits, forming tumors of moderate size and more regular morphology. The arrows indicate the formed tumor lesions.

Two weeks after tumor implantation, confirmed by enhanced CT scan, we successfully implanted tumors in rabbits of group A and group C, for a total of 40 masses in 20 rabbits, with a 100% success rate of tumor implantation. One rabbit in group B had an unsuccessful tumor formation on one side with a success rate of 90%. None of the rabbits developed an infection or died during implantation. Whole-body CT enhancement at two weeks did not reveal tumor metastasis to other parts of the body, such as the liver or lungs. On day 14, we found that the tumors in group A differed significantly in size, ranging from 8.9 to 23.9 mm. 5 cases of metastasis were found along the needle tract. The tumor shapes were round in 2 cases, oval in 3 cases, and irregular in 5 cases. The tumors in group B ranged from 7.3 to 16.1 in diameter, while those in group B were mainly round and oval, with only 3 cases of irregular shapes. Those in group C were uniform in size, ranging from 10.5 to 16.5, while those in group C were mainly round and oval, with only 2 cases of irregular shapes (Fig. 4) (Table 1).

**Table 1:** Percutaneous CT-Guided VX-2 Implantation.

	Group A (Suspension Injection)	Group B (In-vivo tumor scripts)	Group C (In-vitro tumor scripts)
Tumor size (mm, mean ± SD)			
Day 7	8.59 ± 2.77	6.42 ± 1.58	6.91 ± 1.23
Day 14	16.60 ± 4.90	12.24 ± 2.29	13.69 ± 1.80
Tract seeding			
Yes	5	1	1
No	15	18	19
Subcutaneous metastases			
Yes	1	0	0
No	19	19	20
Distant metastases			
Yes	0	0	0
No	20	19	20

### Comparison between group B and group C for tumor size

Since the number of tumor cells in the tumor tissue strips could not be clearly obtained, it was not scientific to compare the tumor size formed by the tissue strips with the cell suspension group. Therefore, the comparison of tumor size was performed only between groups B and C, which used tumor tissue strips, using two independent samples *t*-test.

In this study, independent samples *t*-test was used to determine the tumor size difference formed in groups B and C after 1 and 2 weeks, respectively. One unilateral tumor in group B was not successfully implanted, so a total of 19 masses in size were included in the analysis. The study data did not have significant abnormal values and were nearly normally distributed within each group, while chi-squared. The results showed that the mean tumor size in group B (6.416 ± 1.584) was smaller than that in group C (6.905 ± 1.234) at one week after implantation, with a difference of −0.489 (95% confidence interval −1.408−0.429). The results of the independent samples *t*-test indicated that there was no statistical difference in tumor size between groups B and C at one week (*t* =  − 1.079, *P* > 0.05).

**Figure 5:**
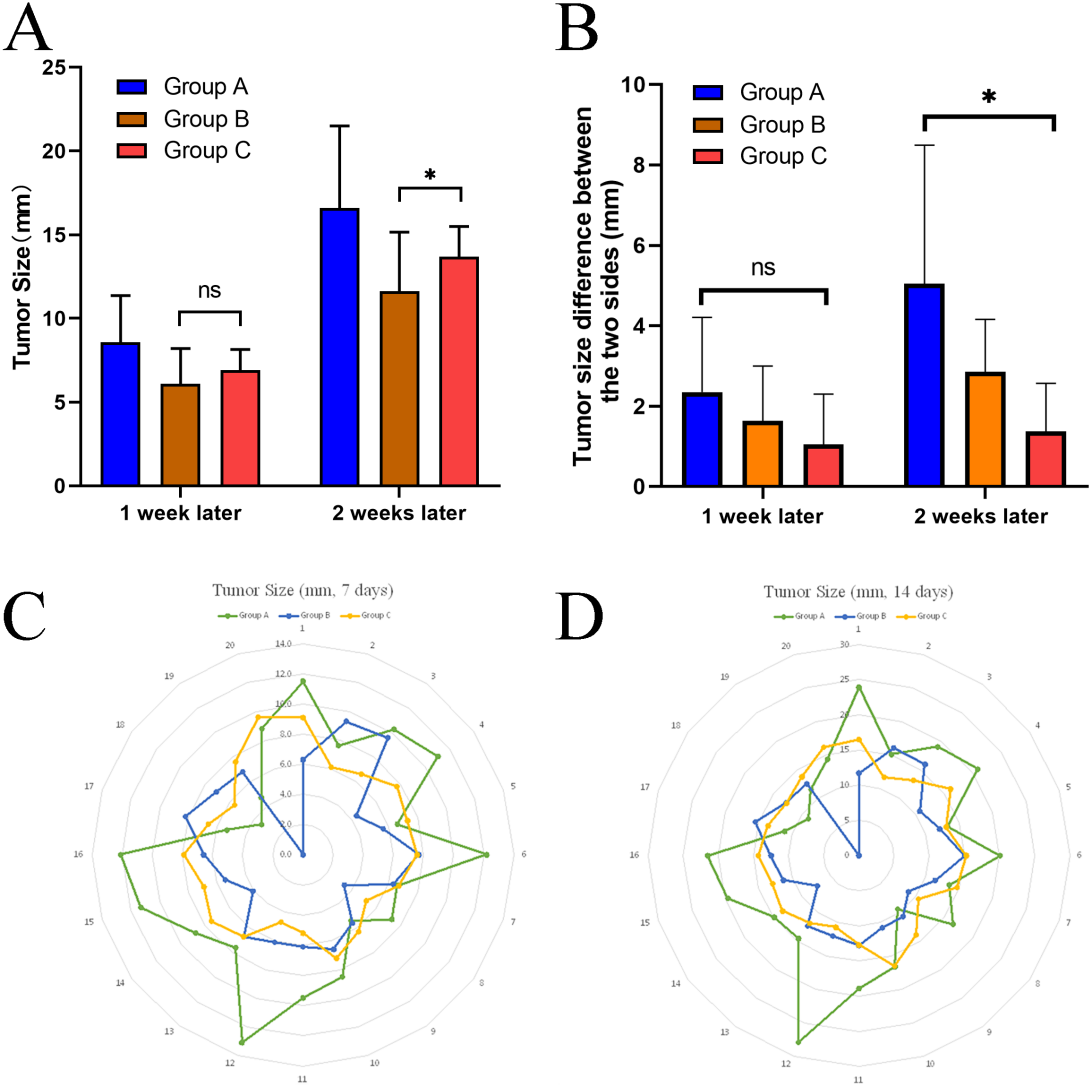
Comparison of size difference between the dual tumor and stability. (A) Differences in tumor size between group B and group C at different time points using a two-sample *t*-test. (B) After two weeks, the size differences between the dual tumor in group A were significantly higher than that in group C, indicating that the size of tumors forming both sides of group A was not as stable as that in group C. (C, D) radar plot showed that the implanted tumors’ size was more stable in group C than in the other two groups at different time points. *P < 0.05, ns, not significant.

At 2 weeks after implantation, the mean tumor size in group B (12.239 ± 2.287) was smaller than in group C (13.685 ± 1.799), with a difference of −1.446 (95% confidence interval −2.777–0.115). The results of the independent samples *t*-test suggested that t = −2.201, *P* < 0.05, indicating that there was a statistical difference in tumor size between groups B and C at two weeks, with group B having a larger tumor size than group C (Fig. 5A).

### Stability of the dual-tumor model in each group

To analyze the stability of tumor size obtained by different inoculation methods, we also calculated the variance of each group after 14 days. It was found that the variance of the data in Group A (Variance =24.04) was significantly higher than that in Group B (Variance =5.229) and Group C (Variance =3.236) (Table 2).

In this study, a one-sample *t*-test was used to determine whether the mean values of tumor differences on both sides of each group differed from the reference values (Table 3). The study data did not have significant outliers and were close to normal distribution. The results showed that when the reference value was set at five mm, the results of the one-sample *t*-test suggested that the difference between the mean value of the difference and the value of the difference test in group A was not statistically significant (*t* = 0.044, *P* = 0.966). On the other hand, groups B and C were statistically significant (*P* < 0.05) from the reference value. When the reference value was set at one mm, the results of the one-sample *t*-test suggested that the mean of the difference values in groups A and B were statistically different from the reference value (*P* < 0.05), while group C was not statistically different from the reference value (*t* = 0.980, *P* = 0.353) (Table 3).

**Table 2:** Tumor growth at different time points.

		Number	Minimum	Maximum	Mean	Std. Deviation	Variance
7 days	Group A	20	3.40	13.10	8.585	2.77456	7.698
	Group B	19	3.40	9.60	6.4158	1.58404	2.509
	Group C	20	4.70	9.60	6.9050	1.23394	1.523
14 days	Group A	20	8.90	27.90	16.5960	4.90350	24.044
	Group B	19	7.30	16.10	12.2389	2.28671	5.229
	Group C	20	10.50	16.50	13.6850	1.79891	3.236

**Table 3:** One-sample *t*-test of the difference in tumor size between the two sides of each group at 2 weeks.

		*t*	df	Sig. (2-tailed)	Mean difference	95% Confidence interval of the difference	
						Lower	Upper
Reference Value = 1 mm	Diff_Group A	3.720	9	.005	4.04800	1.5865	6.5095
	Diff_Group B	4.246	8	.003	1.85111	.8458	2.8564
	Diff_Group C	.980	9	.353	.37000	−.4845	1.2245
Reference value = 2 mm	Diff_Group A	2.801	9	.021	3.04800	.5865	5.5095
	Diff_Group B	1.952	8	.087	.85111	−.1542	1.8564
	Diff_Group C	−1.668	9	.130	−.63000	−1.4845	.2245
Reference value = 5 mm	Diff_GroupA	.044	9	.966	.04800	−2.4135	2.5095
	Diff_GroupB	−4.929	8	.001	−2.14889	−3.1542	−1.1436
	Diff_GroupC	−9.610	9	.000	−3.63000	−4.4845	−2.7755

At one week post-implantation, a one-way ANOVA for the size difference of the dual tumors for each animal in all groups revealed no statistical difference between the different groups (*F* = 1.641, *P* = 0.213).

At two weeks after implantation, Welch’s ANOVA was used to determine whether there was a difference in the size difference of the dual tumors between the groups. There were no abnormal values in the data judged by box plot; each group obeyed normal distribution by Shapiro–Wilk test (*P* > 0.05). By Levene’s chi-square test, it was found that the data of each group did not meet the chi-square. The size difference between dual tumors in all groups was statistically significant (Welch *F* = 6.677, *P* < 0.01). The data are expressed as mean ± standard deviation, and the mean values of the differences for each group were: group A (5.048 ± 3.441), group B (2.851 ± 1.308), and group C (1.370 ± 1.194). The Games-Howell test showed that the mean increase in the size difference between dual tumors from group A to group B was 2.197 (95% CI [−0.938–5.332]), and the size difference was not statistically significant (*P* = 0.189); the mean increase in the size difference between dual tumors from group B to group C was 1.481 (95% CI: [−0.004–2.967]), and the size difference was not statistically significant (*P* = 0.051); from group A to group C, the size difference between dual tumors increased by a mean of 3.678 (95% CI [0.573–6.783]), with a statistically significant difference (*P* < 0.05).

The above results revealed that the size difference between the two sides formed in group A was significantly higher than that in group C, indicating that the dual-tumor model established in group A was less stable than that in group C.

### Pathological features

**Figure 6:**
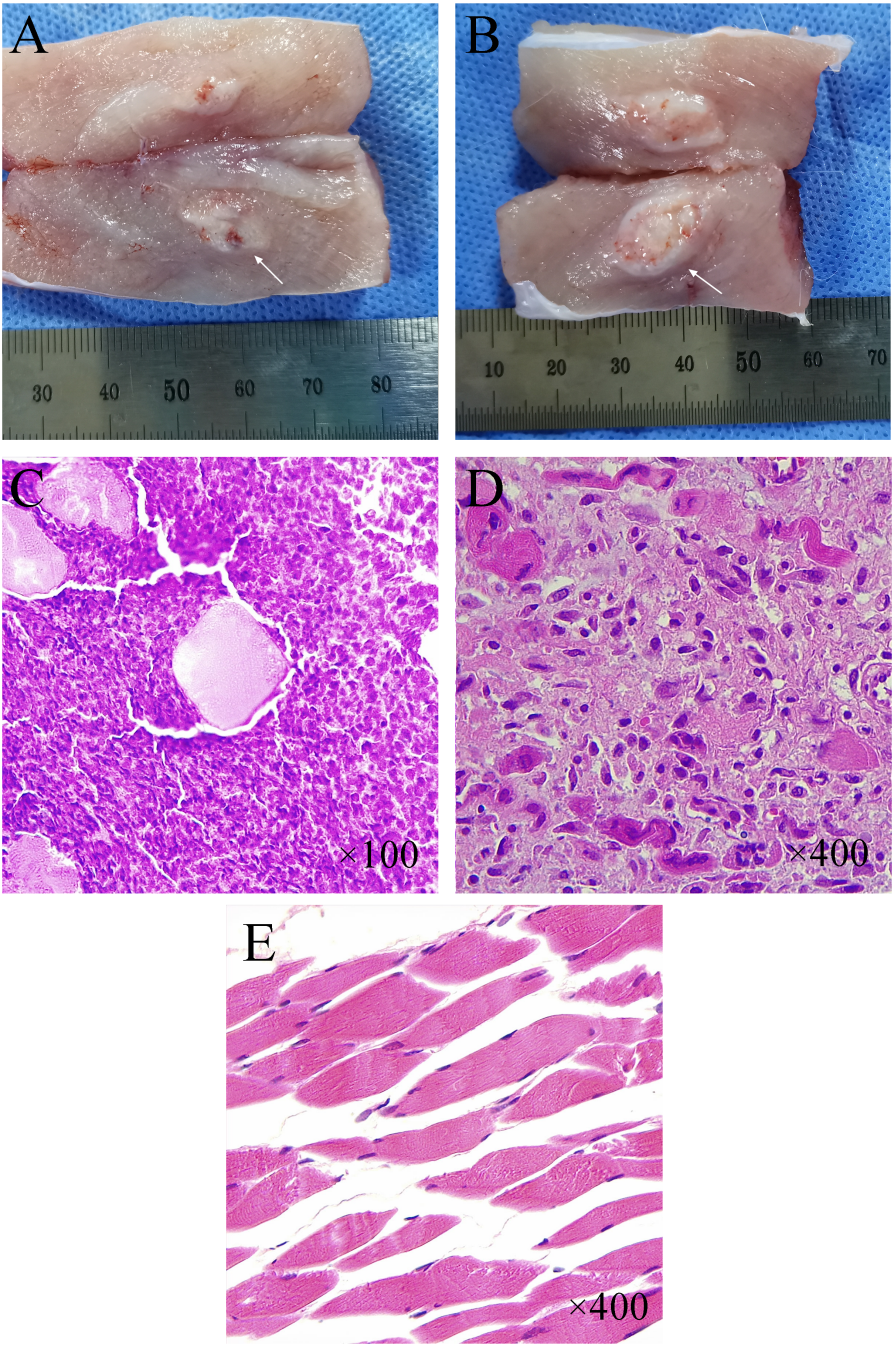
Histological and pathological features of VX-2 tumors. (A, B) Tumor morphology at one week and two weeks after CT-guided tissue strip implantation in group C. (C, D) Pathological histology of tumor tissue (H&E staining) from New Zealand White rabbits with successful tumor inoculation. Under low magnification (×100) (C), densely distributed multifocal infiltrative nests of tumor cells were seen in the tissue. The large pink spot in the center represents the cross-section of residual muscle fibers. Under higher magnification (×400) (D), the nuclei of tumor cells were round or oval with apparent heterogeneity, and nuclear schizophrenia was seen. (E) Regularly arranged long strips of myofibroblasts with nuclei located at the cell margins were seen in the normal muscle tissue surrounding the tumor (×400).

The tumor has a milky white flesh-like appearance, with the pseudo-envelope visible at the edge and clearly demarcated from the muscle tissue (Figs. 6A–6B). Normal muscle tissue can be seen in the tumor tissue. Under lower magnification (×100), multifocal infiltrative carcinoma nests can be seen in the muscle tissue (Fig. 6C). Under higher magnification (×400), tumor cells can be arranged in a cord-like or glandular pattern with polygonal shape, abundant cytoplasm, apparent nuclear heterogeneity, and nuclear division necrotic areas can be seen between tumor tissues (Figs. 6C–6D). HE staining of normal muscle tissue surrounding the tumor showed long strips of myofibroblasts with nuclei located at the edges (×400) (Fig. 6E).

## Discussion

This study establishes a novel paraspinal intramuscular VX-2 dual tumor model. This dual tumor model can be used to study the immune effects of minimally invasive therapy on other parts of the body and the efficacy and mechanisms of minimally invasive cancer treatment combined with other immunotherapies. We used a CT-guided approach to precisely release the tumor in the target area. Also, we compared the results of implantation by tumor suspension injection, in vivo puncture biopsy, and in-vitro direct-view puncture biopsy. It was found that group C, the most stable tumor models obtained by direct in-vitro puncture, were consistent in size and shape and were less prone to subcutaneous or needle tract implantation metastases. Cell suspension injections tend to cause tumor cells to metastasize along with the muscle space and the needle tract, resulting in tumors of varying sizes and shapes. The use of a lipiodol- and Matrigel-containing tumor cell suspension for inoculation has been reported to effectively reduce the spread and metastasis of the tumor suspension during lung cancer modeling (Choi et al., 2017). However, the inoculation site in this study was within the muscle. The anatomical characteristics of the fine fibers and muscle interstitial spaces in muscle tissue led to the possibility of diffusion of the suspension, resulting in irregular tumor morphology or metastasis. In group B, the main problem of in-vivo puncture biopsy is that the tumor size varies, and some implantation failures may occur.

In previous studies, most tumor models have been surgically implanted with tumor fragments or percutaneously injected with tumor suspensions (Chen et al., 2004; Lee et al., 2009). The VX-2 model can be applied in the therapeutic studies of brain tumors. The establishment of brain tumors was usually accomplished by injecting tumor cell suspensions, followed by confirmation of inoculation results by CECT or MRI (Ahmad et al., 2011; Xiao et al., 2009). Chen et al. (2004) used both the intrahepatic injection of VX-2 suspension and surgical incision followed by implantation of tumor fragments to establish a rabbit liver cancer model. They found that the liver’s direct implantation of VX-2 tumor fragments was more effective than the injection of VX-2 tumor cell suspension into the liver. Three rabbits showed no tumor growth in the tumor cell suspension group, and ten rabbits showed signs of extrahepatic leakage and tumor implantation. In the other group, all but one rabbit showed tumor growth and no tumor implantation metastases. The disadvantages of the surgical approach to tumor implantation are also apparent. First, the surgical procedure is time-consuming and resource-consuming. Second, there are many surgical complications, including infection and bleeding, leading to higher mortality rates in severe cases (Chen et al., 2004). In the construction of hepatocellular carcinoma models, the dropping of tumor fragments can cause ectopic tumor implantation. Tong et al. constructed a modified tumor block implantation model to avoid this complication. They pre-implanted the tumor block embedded in the gelatin sponge into the left lobe of the liver. They then sealed it with a gelatin sponge to establish the VX-2 hepatocellular carcinoma model. This approach significantly reduced the occurrence of tumor leakage and ectopic dissemination (Tong et al., 2018).

Another method that has been evolved is the ultrasound-guided inoculation method (White et al., 2015). White et al. successfully implanted tumor fragments into the liver of 27 rabbits percutaneously under ultrasound guidance. Tumor formation was successful in 24 of these rabbits. Among them, only 2 cases of tract seeding occurred. Lee et al. compared the success rates and complications of ultrasound-guided and open procedures to establish a hepatocellular carcinoma model. They found that the implantation success rate of ultrasound-guided implantation was comparable to that of an open procedure. Still, the rate of tract seeding was higher than that of the open procedure (Lee et al., 2009). Compared with ultrasound guidance, CT’s main advantages are fast imaging speed, high-density resolution, and clear tomographic anatomical relationships, which facilitate our more precise localization and thus obtain a bilateral tumor model with relatively symmetrical location and more favorable observation.

In the present study, a dual tumor model was developed because in some experimental designs, a rabbit two-focal tumor model is needed, in which two tumors are treated with different interventions, such as radiofrequency ablation on one side of the tumor, with the other side as a control, or chemical ablation with different types or doses of drugs on both sides of the tumor. Besides, the effect of one tumor receiving the intervention on the other can be analyzed using a two-sided tumor model. There are few reports of such bilateral tumor models. Only Ahn et al. (2018) used a bilateral intramuscular tumor model to investigate the effect of CKD-516 on changes in blood flow and metabolic parameters in PET/MRI. However, they used the cell suspension injection method, and some of the tumor models can be seen to have implantation metastasis from the MR images.

As for the location of the tumor within the paraspinal muscles bilaterally, there are several considerations. First, the tissue structure here is well fixed, making it easy to puncture and locate the tumor and is not easily influenced by the respiratory and heartbeat. Secondly, because the back muscles are thick and robust, the trocar can be easily fixed, reducing the ectopic implantation caused by the trocar’s movement. Thirdly, it is far away from the abdominal organs, so it is less prone to abdominal transfer. After anesthesia, the rabbit is placed in the prone position, which can fully expose the surgical area and facilitate experimental procedures such as cryoablation, radiofrequency, and drug injection.

This study compared the effects of tumor cell suspension and tissue strip modeling. We concluded that the tissue strip is the better way to inoculate in muscle tissue based on the results. Sources of tumor cell suspensions usually include tumor cells cultured in vitro and tumor tissue cell suspensions made by homogenizing and filtering fresh tumor tissue (Li et al., 2018). With more passaging of VX-2 cells for in vitro culture, the enzymatic digestion method disrupts the relationship between the tumor cells and the parental cells, making the cells less invasive and affecting the implantation’s success rate. In contrast, tumor cell suspensions prepared from fresh tumor tissues are highly adaptable and resistant to the body’s immunity, resulting in a higher tumorigenic rate than tumor cell suspensions cultured in vitro. In the present study, we found that needle tract seeding occurred more frequently using the syringe-injected suspension method. In contrast, the needle tract implantation rate was relatively low for the method using the punctured needle sheath. This is mainly due to the difference in the implanted tumor’s morphology, where cell suspensions are more likely to spread along the extracted needle tract and thus lead to tract seeding. In contrast, tissue strips are less likely to develop.

Different studies have different protocols regarding the volume and total cell count of the injected tumor suspension. Ueki et al. (2017) used 0.2 ml of tumor suspensions totaling 5 ×10^6^ cells to construct a tumor model for radiofrequency ablation of the lung. Ahmad et al. (2011) established a resectable brain tumor model, and the number of cells they used was 1.8 × 10^3^. Ahn et al. (2018) established a tumor model within the back muscle with 0.2 ml of tumor suspension to determine whether CKD-516 produces significant changes in metabolic parameters in PET/MRI. Chen et al. (2004) established a liver cancer model using a tumor suspension containing 1 ×10^6^ cells with a volume of 0.1 ml. In this study, we used a dosage of 0.2 ml of a total of 5 ×10^5^ cells in suspension, which was determined based on the experience of the previous work in our orthopedic institute. Similar to the above study, the tumors we obtained by suspension injection method varied in size. We considered the total number and volume of injected cells largely dependent on the experiment’s purpose. And a too small injection volume may result in an excessive proportion of tumor cells remaining in the syringe and affect the actual amount implanted.

We obtained more stable results in the present study using an in vitro puncture biopsy to take tissue strips. Compared with cell suspension, tissue strip inoculation can ensure the concentration of tumor cell growth and inter-tissue interaction, reduce the isolation time of most tumor cells, and become a barrier for tumor cells to resist the body’s immunity and enhance the adaptability to the new environment, so the tumorigenic rate is higher. The tissue strips obtained by in-vitro direct vision puncture can ensure that each strip has approximately the same volume of tissue and can effectively avoid normal muscle tissue and necrotic tumor tissue, thus ensuring the implantation of approximately equal amounts of tumor cells. Therefore, the tumor model we obtained in group C had a high survival rate and had little difference in size, making it an ideal bilateral tumor model that can be applied in preclinical studies. The uniformity of tumor size between the two sides also represents the stability of the model. We did not directly compare the tumor sizes of the left and right sides because the choice of left or right side should not have an impact on the results for the implantation method. To reflect the uniformity of tumor size between the two sides, we calculated the variance of the difference in tumor size between the two sides (absolute value) within each group. Finally, we found that the variance of group C was significantly smaller than that of groups A and B, reflecting the uniformity of tumor size on both sides and the stability of the recommended model in group C. Besides, tissue strips should be pushed into the muscle tissue using a needle core with a flattened tip during tumor tissue strip implantation. Tumor strips are easily cut and adhere to them when pushed with a sharp needle core. After flattening the needle tip, the mating needle core can be two mm longer than the needle sheath and should be sufficient to push the tumor fragments intact into the muscle. After pushing, the tissue strips will be placed in clusters in the inoculation area, facilitating consistent round-like tumors.

In addition, the tumor implantation method established in this study can be applied to the modeling of various site-specific tumors throughout the body, including lung cancer, liver cancer and bone tumors. Especially in the construction of bone tumor models, our method can precisely implant tumor tissue strips into bone to form a stable tumor model while ensuring the integrity of bone structure. An ongoing study on radiofrequency ablation of lung nodules in our institution has used this method to establish isolated solid tumor models in the lung, and has preliminary obtained accurate and stable tumor models. More studies are needed in the future to verify the feasibility and stability of our method.

There are several limitations in this study. First, the size of tumors formed by the cell suspension method and the tumor tissue strip method cannot be directly compared because the total number of cells implanted in the tumor tissue strip cannot be determined. However, it is feasible to compare the tumor formation rate, metastasis rate, and dual tumors’ stability for each method separately. Secondly, due to hardware equipment and financial limitations, the tumor’s imaging could not be selected for sharper MR enhancement. MR imaging can better show the tumor parenchyma, necrosis, and surrounding edema. However, the imaging speed of MR is slower than CT. Thirdly, the contrast agent was manually injected through the ear margin vein. There might be a slight deviation between the enhanced imaging and the actual size of the tumor. Besides, confounding factors such as the batch of animals, weight, duration, and sequence of operations were not considered in analyzing the results.

## Conclusions

This study attempted to establish an accurate and stable intramuscular dual tumor model by comparing three different inoculation methods. By comparing the tumorigenic rate and the consistency of the dual tumor size of the three methods, we found that the technique of using a puncture biopsy needle to harvest tumor tissue strips in vitro followed by CT-guided implantation of the tissue strips through a needle sheath has a high tumorigenesis rate and a stable model, and is an ideal dual tumor model for application in preclinical studies such as minimally invasive surgery or observation of local drug therapy.

##  Supplemental Information

10.7717/peerj.11536/supp-1 Supplemental Information 1 Tumor size measurements at 7 days

10.7717/peerj.11536/supp-2 Supplemental Information 2 Tumor size measurements at 14 days

10.7717/peerj.11536/supp-3 Supplemental Information 3 The ARRIVE guidelines 2.0: author checklist

## Additional Information and Declarations

### Data Availability

The following information was supplied regarding data availability: The raw measurements are available in the Supplemental Files.
